# pH-responsive Virus-like Nanoparticles with Enhanced Tumour-targeting Ligands for Cancer Drug Delivery

**DOI:** 10.1038/srep37891

**Published:** 2016-11-24

**Authors:** Roya Biabanikhankahdani, Noorjahan Banu Mohamed Alitheen, Kok Lian Ho, Wen Siang Tan

**Affiliations:** 1 Department of Microbiology, Faculty of Biotechnology and Biomolecular Sciences, Universiti Putra Malaysia, 43400 UPM Serdang, Selangor, Malaysia; 2 Department of Cell and Molecular Biology, Faculty of Biotechnology and Biomolecular Sciences, Universiti Putra Malaysia, 43400 UPM Serdang, Selangor, Malaysia; 3 Institute of Bioscience, Universiti Putra Malaysia, 43400 UPM Serdang, Selangor, Malaysia; 4 Department of Pathology, Faculty of Medicine and Health Sciences, Universiti Putra Malaysia, 43400 UPM Serdang, Selangor, Malaysia

## Abstract

Multifunctional nanocarriers harbouring specific targeting moieties and with pH-responsive properties offer great potential for targeted cancer therapy. Several synthetic drug carriers have been studied extensively as drug delivery systems but not much information is available on the application of virus-like nanoparticles (VLNPs) as multifunctional nanocarriers. Here, we describe the development of pH-responsive VLNPs, based on truncated hepatitis B virus core antigen (tHBcAg), displaying folic acid (FA) for controlled drug delivery. FA was conjugated to a pentadecapeptide containing nanoglue bound on tHBcAg nanoparticles to increase the specificity and efficacy of the drug delivery system. The tHBcAg nanoparticles loaded with doxorubicin (DOX) and polyacrylic acid (PAA) demonstrated a sustained drug release profile *in vitro* under tumour tissue conditions in a controlled manner and improved the uptake of DOX in colorectal cancer cells, leading to enhanced antitumour effects. This study demonstrated that DOX-PAA can be packaged into VLNPs without any modification of the DOX molecules, preserving the pharmacological activity of the loaded DOX. The nanoglue can easily be used to display a tumour-targeting molecule on the exterior surface of VLNPs and can bypass the laborious and time-consuming genetic engineering approaches.

In recent years, nano-scale carriers with a pH-triggered release mechanism have attracted increasing attention for the development of controlled drug delivery systems. When an intracellular pH-triggered drug release carrier is incorporated with a tumour-targeting ligand, this multifunctional nanocarrier can recognize tumour cells and release the encapsulated drug at tumour sites in a controlled manner^1^,^2^. A variety of nanomaterials responding to pH stimuli, such as liposomes, micelles, polymeric and prodrug nanoparticles, have been synthesised and developed as effective drug delivery systems^3^,^4^,^5^,^6^. However, not much effort has gone toward developing a pH-responsive drug delivery system based on virus-like nanoparticles (VLNPs). VLNPs are composed of natural biological building blocks, and they exhibit great potential for revolutionizing medicine as new noninfectious nanocarrier platforms^7^,^8^,^9^.

Hepatitis B core antigen (HBcAg) self-assembles into VLNPs, which have been shown to be some of the most powerful protein engineering tools employed to display immunogens and cell-targeting peptides, as well as for the packaging of genetic materials^10^. An HBcAg mutant, namely truncated HBcAg (tHBcAg), also self-assembles into icosahedral nanoparticles of approximately 35 nm, which can be used to package green fluorescent protein (GFP)^11^,^12^,^13^. A liver-specific ligand (preS1) fused at the N-terminus of the tHBcAg was demonstrated to deliver fluorescein molecules into HepG2 cells^14^. These discoveries have paved the way for exploiting tHBcAg nanoparticle as targeted drug delivery systems.

Displaying folic acid (FA) on VLNPs is a popular strategy to enhance specific uptake by tumour cells through folate receptor (FR)-mediated endocytosis^15^,^16^. However, conjugation of FA directly onto VLNPs may cause inaccessibility of FA molecules to the FR^17^. Conjugation of FA to a sufficiently long PEG-chain has been shown to be an effective way of targeting nano-emulsions and VLNPs to cancer cells^17^,^18^. In this study, we applied an alternative and relatively simple strategy for the preparation of surface-modified VLNPs for cancer-targeting drug delivery. A pentadecapeptide containing the capsid binding sequence (nanoglue), which interacts specifically at the spikes of tHBcAg nanoparticles, was employed to display the tumour-targeting molecules (Fig. 1). FA molecules were conjugated to the free Lys residues at the N-terminal end of the pentadecapeptide bound on tHBcAg nanoparticles. In this manner, the FA molecules extend flexibly away from the nanoparticle, and their exposure to target FRs on the surface of cancer cells is maximized. This should enhance the tumour-targeting activity of tHBcAg nanoparticles loaded with doxorubicin (DOX).

DOX is a potent drug commonly used in the treatment of numerous types of cancers, including breast, lung, ovarian and colorectal cancers^19^. However, its use is restricted by low solubility and serious side effects, including congestive heart failure^20^,^21^. Therefore, it is important to establish a specific drug delivery system to cancer cells using FA-conjugated tHBcAg nanoparticles, and reduce the side effects on normal cells. DOX has a small molecular mass of approximately 545 Da, which makes it difficult to load and be retained inside VLNPs^22^. To package DOX inside tHBcAg nanoparticles and to release the drug in a controlled manner, polyacrylic acid (PAA) was mixed with DOX and loaded into the tHBcAg nanoparticles during the reassociation of the particles. The pKas of PAA and DOX are 4.8 and 8.6, respectively^23^,^24^, and an electrostatic interaction takes place between the negatively charged PAA and positively charged DOX at pH 7.4, and this interaction is reversible at low pH. Thus, at the physiological pH of normal tissues, DOX is retained in tHBcAg nanoparticles, and it is only released when the nanoparticles reach extracellular tumour tissues or intracellular endosomes with a pH approximately 5–5.5. In this study, pH-responsive tHBcAg nanoparticles displaying FA molecules were demonstrated to improve the uptake of DOX by colorectal cancer cells, and the drug was released in a controlled manner.

## Results

### Synthesis and characterization of FA-tHBcAg nanoparticles

**Figure 1:**
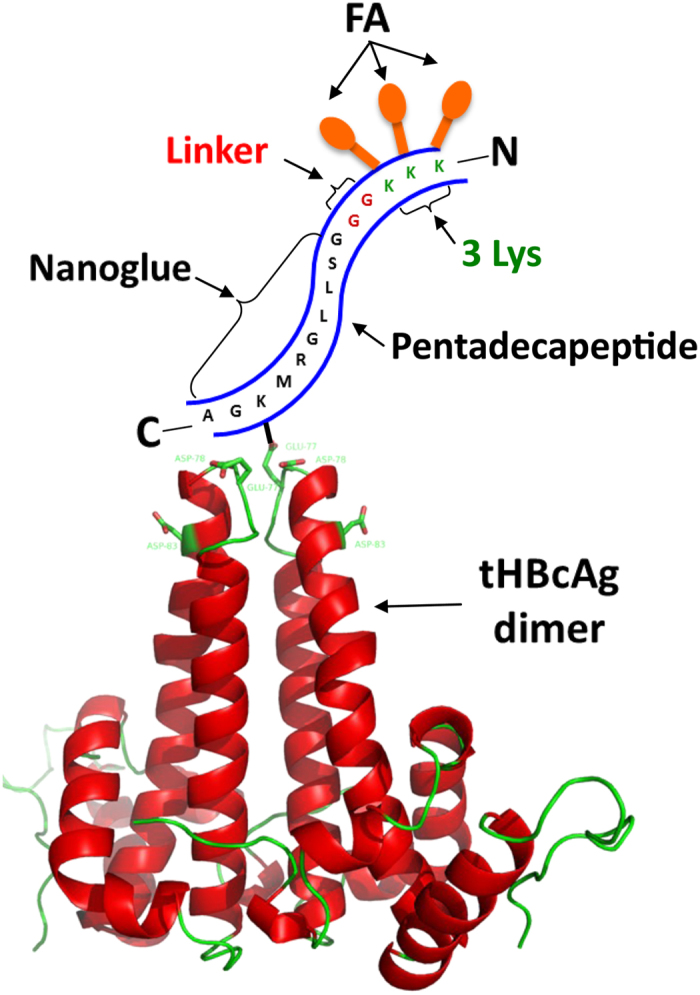
Displaying of folic acid molecules at the tip of a tHBcAg dimer using the nanoglue. A pentadecapeptide containing the nanoglue, linker and 3 Lys residues interacts with a tHBcAg dimer. The nanoglue binds specifically at the tip of the tHBcAg dimer and its Lys is cross-linked to Asp or Glu using EDC and Sulfo-NHS. Folic acid (FA) molecules are then conjugated to the free Lys residues at the N-terminus of the pentadecapeptide. The tHBcAg dimer self-assembles into nanoparticles.

**Figure 2:**
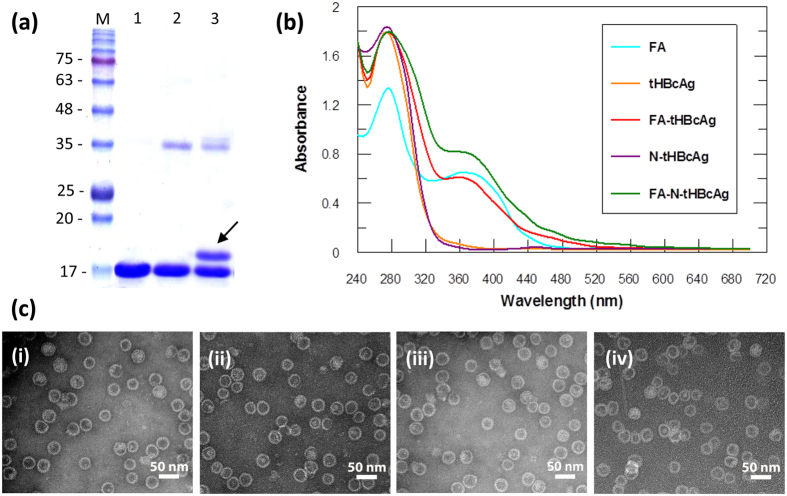
Conjugation of folic acid to tHBcAg nanoparticles using the nanoglue concept. (**a**) An SDS-polyacrylamide gel of the tHBcAg nanoparticles cross-linked with the pentadecapeptide containing the nanoglue. Lane M, molecular mass markers (kDa); lane 1, tHBcAg; lane 2, tHBcAg plus cross-linkers; and lane 3, tHBcAg plus the pentadecapeptide and cross-linkers. The arrow shows a shifted band of approximately 1 kDa above the 17 kDa tHBcAg. (**b**) Conjugation of folic acid (FA) to tHBcAg. Spectra of FA, tHBcAg nanoparticles (tHBcAg), tHBcAg nanoparticles cross-linked with pentadecapeptide (N-tHBcAg), FA-conjugated tHBcAg nanoparticles (FA-tHBcAg), and FA-conjugated tHBcAg nanoparticles using the nanoglue (FA-N-tHBcAg). (**c**) Electron micrographs of tHBcAg nanoparticles. Nanoparticles formed by tHBcAg (i), N-tHBcAg (ii), FA-tHBcAg (iii), and FA-N-tHBcAg (iv) were stained with uranyl acetate and observed via TEM. White bars indicate 50 nm.

To increase the accessibility of FA to the FR, nanoglue was used to display FA molecules at the spikes of tHBcAg nanoparticles (Fig. 1). A pentadecapeptide (KKKGGGSLLGRMKGA) containing 3 Lys residues at the N-terminus was synthesised and cross-linked to tHBcAg nanoparticles using Sulfo-NHS and EDC. The tHBcAg monomer shifted approximately 1 kDa on an SDS-polyacrylamide gel, demonstrating that the peptide was cross-linked to the monomer (Fig. 2a). The cross-linked tHBcAg nanoparticles were then conjugated with FA by using Sulfo-NHS and EDC, and the absorbance at wavelength 240–700 nm was measured. Conjugation of FA to tHBcAg using the nanoglue concept (FA-N-tHBcAg) exhibited a greater A_360_ value compared with conjugation of FA to tHBcAg (FA-tHBcAg) directly (Fig. 2b). The conjugation efficiency (CE) of FA was 6.1 ± 0.3% and 12.4 ± 0.5%, amounting to approximately 461 and 953 FA molecules conjugated to each FA-tHBcAg and FA-N-tHBcAg nanoparticle, respectively. FA:tHBcAg stoichiometry was determined to be 2:1 for FA-tHBcAg and 4:1 for FA-N-tHBcAg. This is because FA can be conjugated to the Lys residues of the pentadecapeptide and tHBcAg nanoparticles. Transmission electron micrographs showed that cross-linking of the nanoglue followed by conjugation of FA did not have an adverse effect on the spherical structure of the tHBcAg nanoparticles (Fig. 2c). To verify that FA molecules were indeed conjugated to Lys residues at the N-terminus of the pentadecapeptide, Lys-7 and Lys-96 of tHBcAg were first conjugated with NHS-fluorescein. The fluorescein-labelled tHBcAg nanoparticles (ftHBcAg) were then cross-linked with the pentadecapeptide and conjugated with FA (Supplementary Figure S1). A_360_ showed that FA was conjugated to the pentadecapeptide, and A_500_ demonstrated that tHBcAg was cross-linked with fluorescein molecules.

**Figure 3:**
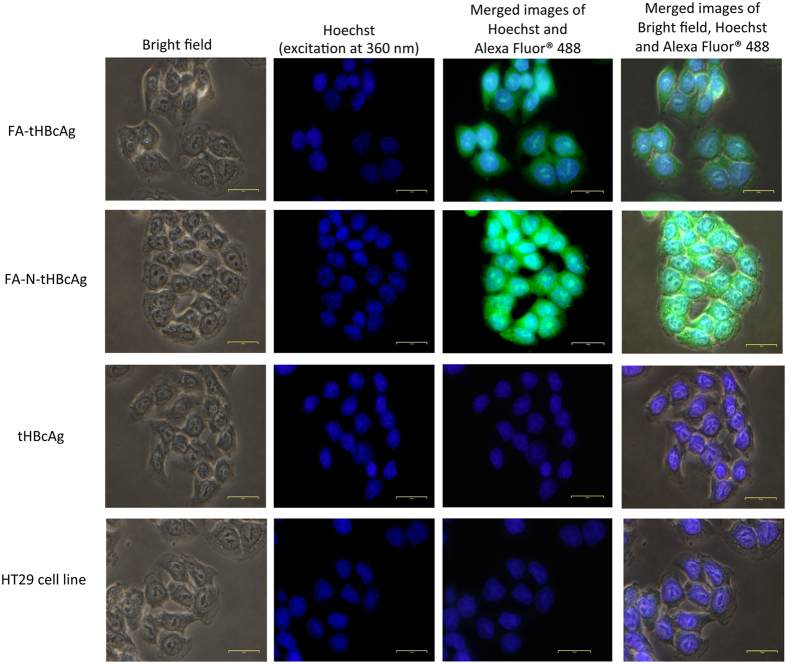
Internalization of tHBcAg nanoparticles conjugated with folic acid into HT29 cells. The cells were incubated with different tHBcAg nanoparticles (25 μg) for 16 h at 37 °C. The cells were then permeabilized with methanol and the internalized tHBcAg particles were detected by rabbit anti-tHBcAg serum, followed by anti-rabbit antibody conjugated to Alexa Fluor^®^ 488. Nuclei were stained with Hoechst (blue). Non-transfected HT29 cells were used as a negative control. The samples are labelled on the left: Folic acid (FA)-conjugated tHBcAg nanoparticles (FA-tHBcAg), FA-conjugated tHBcAg nanoparticles using the nanoglue (FA-N-tHBcAg) and tHBcAg nanoparticles (tHBcAg).

The internalization of FA-conjugated tHBcAg nanoparticles into HT29 cells was studied by immuno-fluorescence microscopy, in which tHBcAg was detected by rabbit anti-tHBcAg antibody followed by anti-rabbit IgG conjugated to Alexa Fluor^®^ 488. HT29 cells were used because they over-express the FR^25^. Figure 3 shows that the FA-tHBcAg nanoparticles translocated into HT29 cells, and the cells fluoresced. The green fluorescent signal increased considerably when FA-N-tHBcAg nanoparticles were used to transfect the cells (Fig. 3). This indicates that conjugation of FA to the pentadecapeptide bound to tHBcAg *via* the nanoglue enhanced the uptake of tHBcAg nanoparticles by HT29 cells. FA-tHBcAg and FA-N-tHBcAg nanoparticles accumulated in the cytoplasm of HT29 cells and no green fluorescent signal was detected in the nuclei of cells that were labelled with Hoechst (Fig. 3). This demonstrates that FA mediates the internalization of tHBcAg particles into the cytoplasm of HT29 cells but not into the nucleus. tHBcAg nanoparticles alone (tHBcAg) did not internalize HT29 cells.

### Packaging of doxorubicin by tHBcAg nanoparticles

**Figure 4:**
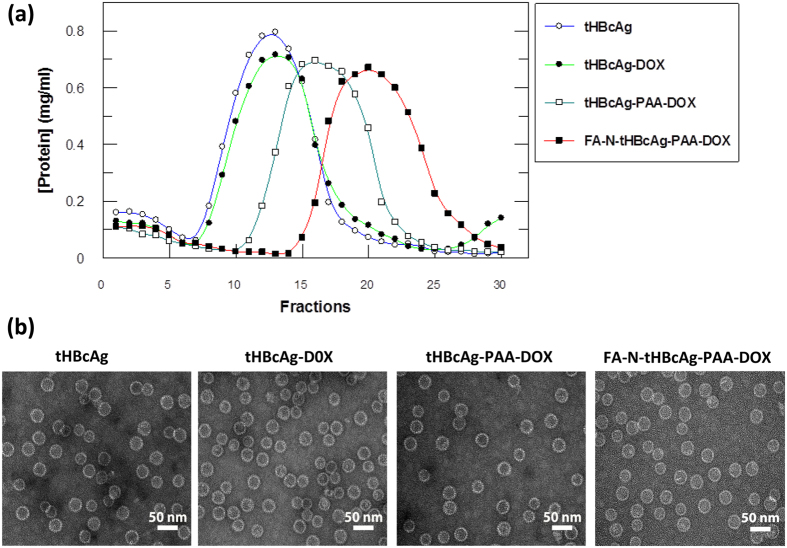
Packaging of doxorubicin by tHBcAg nanoparticles. (**a**) Sucrose density gradient ultracentrifugation of different tHBcAg nanoparticle packaging samples. tHBcAg nanoparticles containing doxorubicin (DOX) were separated by ultracentrifugation on sucrose gradients (8–40%). The total protein in each fraction (400 μL) was determined by a Bradford assay. Packaging of DOX with tHBcAg nanoparticles (tHBcAg-DOX), tHBcAg nanoparticles loaded with PAA-DOX (tHBcAg-PAA-DOX), and FA-conjugated tHBcAg nanoparticles using the nanoglue and loaded with PAA-DOX (FA-N-tHBcAg-PAA-DOX). tHBcAg nanoparticles (tHBcAg) were used as a negative control. (**b**) Electron micrographs of the tHBcAg nanoparticles from different packaging samples. The samples are labelled on top of the micrographs. All the samples assembled into spherical structures. White bars indicate 50 nm.

The method of dissociation and association^13^ was used to package DOX and PAA inside the tHBcAg nanoparticles. Figure 4a shows the migration profiles of different packaging samples in a sucrose gradient. In the absence of cargo, the tHBcAg nanoparticles migrated into the gradient and accumulated in fractions 7–18. The migration profile is in accord with that reported by Tan *et al*.^26^. Packaging of DOX in the absence of PAA (tHBcAg-DOX) did not result in any differences in the migration profile of the nanoparticles compared with that of tHBcAg (Fig. 4a). This demonstrates that DOX alone cannot be packaged by tHBcAg nanoparticles. When PAA and DOX were loaded, the tHBcAg-PAA-DOX nanoparticles migrated faster in the gradient, and the peak shifted to a higher density, demonstrating the presence of denser nanoparticles (fractions 13–22).

The tHBcAg nanoparticles loaded with PAA-DOX (tHBcAg-PAA-DOX) were then conjugated with FA using the nanoglue concept (FA-N-tHBcAg-PAA-DOX). Sucrose density gradient centrifugation of the FA-N-tHBcAg-PAA-DOX nanoparticles showed that the particles accumulated in fractions 16–27 (Fig. 4a). The sedimentation rate of this sample was higher than that of tHBcAg-PAA-DOX nanoparticles, indicating that tHBcAg nanoparticles loaded with PAA-DOX were cross-linked with the pentadecapeptide and conjugated with FA. Electron microscopic analysis revealed that all of the packaging samples and FA-conjugated products assembled into icosahedral structures that were morphologically similar to the tHBcAg nanoparticles (Fig. 4b).

**Figure 5:**
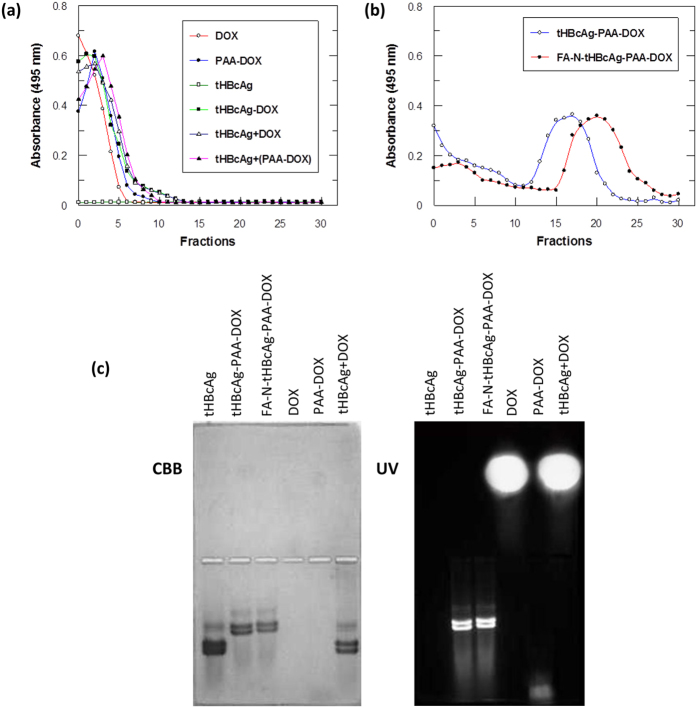
Identification of doxorubicin packaged in tHBcAg nanoparticles. Packaging samples were separated on sucrose gradients (8-40%) and fractionated (400 μL), and A_495_ was measured to detect doxorubicin. (**a**) Doxorubicin (DOX), doxorubicin with PAA (PAA-DOX), packaging of DOX with tHBcAg nanoparticles (tHBcAg-DOX), tHBcAg particles incubated with DOX without the packaging process (tHBcAg + DOX), and tHBcAg incubated with PAA-DOX without the packaging process [tHBcAg + (PAA-DOX)] stayed on top of the sucrose gradient after centrifugation. tHBcAg nanoparticles (tHBcAg) were not detected at A_495_. (**b**) DOX packaged together with PAA in tHBcAg nanoparticles migrated into sucrose gradients. tHBcAg nanoparticles loaded with PAA-DOX (tHBcAg-PAA-DOX) and folic acid (FA)-conjugated tHBcAg nanoparticles using the nanoglue and loaded with PAA-DOX (FA-N-tHBcAg-PAA-DOX) migrated into the sucrose gradient, and the packaged DOX was detected at A_495_. (**c**) Native agarose gel electrophoresis of tHBcAg nanoparticles loaded with DOX. The same gel was stained with Coomassie brilliant blue (CBB) and visualized under ultraviolet (UV) illumination.

The presence of DOX in tHBcAg nanoparticles was determined with a spectrophotometer at A_495_. The packaging samples were separated on sucrose gradients (8–40%), fractionated (400 μL), and A_495_ was determined. The results revealed that the free DOX, PAA-DOX, and DOX from tHBcAg-DOX samples were retained in the top fractions of the gradients (Fig. 5a). tHBcAg nanoparticles were not detected at A_495_. In contrast, tHBcAg-PAA-DOX and FA-N-tHBcAg-PAA-DOX were detected in fractions 12–21 and 16–26, respectively (Fig. 5b). Packaging of DOX together with PAA inside the tHBcAg nanoparticles increased the density of the nanoparticles, which resulted in a shift of the migration profile to denser fractions.

To rule out the likelihood that the increased density of tHBcAg-PAA-DOX nanoparticles was caused by attachment of DOX on the surface of tHBcAg nanoparticles, the nanoparticles were incubated with DOX (tHBcAg + DOX) and PAA-DOX [tHBcAg + (PAA-DOX)] overnight (without the dissociation and association process) and analysed with sucrose density gradient centrifugation (Fig. 5a). A_495_ readings revealed that the DOX and PAA-DOX stayed on the top of the sucrose gradients, which ruled out the possibility that they were bound on the surface of tHBcAg nanoparticles and migrated together in the sucrose gradient.

### Native agarose gel electrophoresis of tHBcAg nanoparticles loaded with doxorubicin

Native agarose gel electrophoresis was used to confirm the presence of DOX inside tHBcAg nanoparticles. The agarose gel stained with Coomassie brilliant blue (CBB) showed that tHBcAg, tHBcAg-PAA-DOX and FA-N-tHBcAg-PAA-DOX nanoparticles migrated to the anode (Fig. 5c). The tHBcAg-PAA-DOX and FA-N-tHBcAg-PAA-DOX nanoparticles migrated slower compared with the tHBcAg nanoparticles (Fig. 5c). The tHBcAg nanoparticles mixed with DOX (without the dissociation and association process; namely tHBcAg + DOX) also migrated to the anode. When the gel was exposed to UV light, the tHBcAg-PAA-DOX and FA-N-tHBcAg-PAA-DOX bands fluoresced (Fig. 5c), indicating that DOX was packaged in these nanoparticles. The DOX incubated with tHBcAg (tHBcAg + DOX) migrated to the cathode, similar to the free DOX that was included as a control (Fig. 5c). As the tHBcAg nanoparticles and DOX migrated to different directions, this demonstrated that DOX did not bind on the surface of tHBcAg nanoparticles.

The loading efficiency (LE) of DOX was 13.4 ± 0.4% and 12.9 ± 0.5%, amounting to approximately 983 and 946 DOX molecules packaged in each tHBcAg-PAA-DOX and FA-N-tHBcAg-PAA-DOX nanoparticle, respectively.

### *In vitro* release of doxorubicin from tHBcAg-PAA-DOX, FA-tHBcAg-PAA-DOX and FA-N-tHBcAg-PAA-DOX nanoparticles

**Figure 6:**
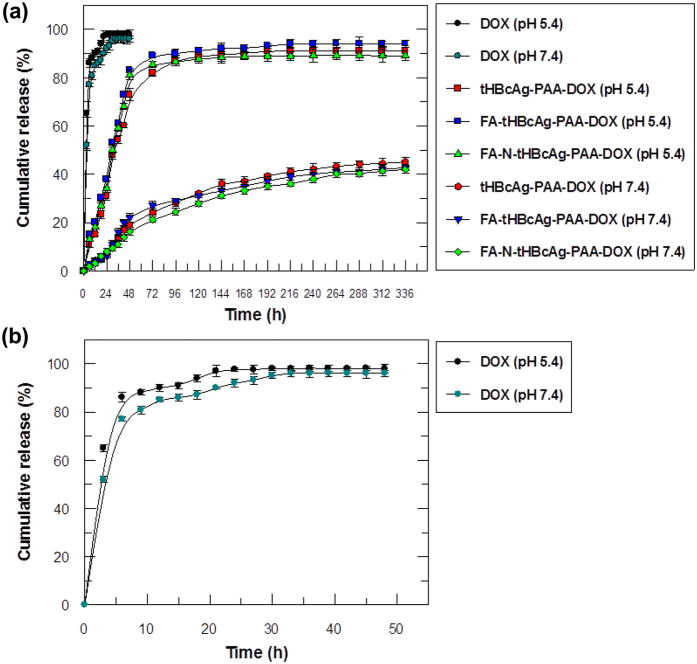
Cumulative release profile of doxorubicin packaged in tHBcAg nanoparticles in different pH conditions. **(a)** Release profile of free doxorubicin (DOX), tHBcAg nanoparticles loaded with PAA-DOX (tHBcAg-PAA-DOX), folic acid (FA)-conjugated tHBcAg nanoparticles loaded with PAA-DOX (FA-tHBcAg-PAA-DOX), and FA-conjugated tHBcAg nanoparticles using the nanoglue and loaded with PAA-DOX (FA-N-tHBcAg-PAA-DOX) at pH 5.4 and 7.4. Approximately 80% of the DOX packaged in tHBcAg nanoparticles was released after 2 days at pH 5.4. However, at pH 7.4, only approximately 19% of the DOX was released from the particles after 2 days, demonstrating slow release of DOX at physiological pH. **(b)** Enlargement of the free DOX release profile. More than 80% of the free DOX was released after 6 h, at pH 5.4 and pH 7.4. Data are expressed as the mean ± standard deviation (n = 3).

An *in vitro* drug release experiment was performed under simulated physiological (pH 7.4, 37 °C)^27^ and tumour tissue and endosomal conditions (pH 5.4, 37 °C)^28^ at various time points. The drug release profiles of the tHBcAg-PAA-DOX, FA-tHBcAg-PAA-DOX and FA-N-tHBcAg-PAA-DOX particles were compared with free DOX (Fig. 6). After 6 h of incubation, approximately 80% of the free DOX was released into the receptor chamber, and almost all of it was released within 24 h (Fig. 6a and b). No significant difference was observed in the cumulative release rate of free DOX in the different pH solutions. In contrast, tHBcAg-PAA-DOX, FA-tHBcAg-PAA-DOX and FA-N-tHBcAg-PAA-DOX nanoparticles did not exhibit any significant release of DOX in the initial hours, resulting in more sustained DOX release patterns over the subsequent 2 days at pH 5.4 (Fig. 6a). In addition, the cumulative release of DOX from tHBcAg-PAA-DOX, FA-tHBcAg-PAA-DOX and FA-N-tHBcAg-PAA-DOX nanoparticles was significantly higher at pH 5.4 (P < 0.01) compared with pH 7.4, indicating that DOX is released more efficiently and in a controlled manner in tumour tissue and endosomal conditions.

### Uptake of tHBcAg nanoparticles loaded with doxorubicin by colorectal cancer cells

**Figure 7:**
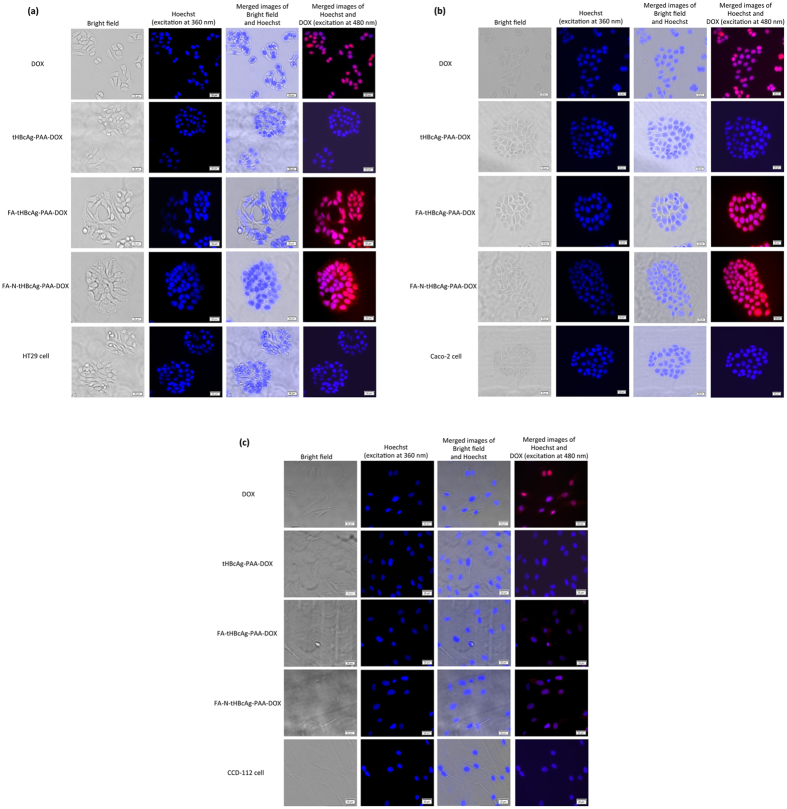
Live cell imaging analysis of the delivery of doxorubicin by tHBcAg nanoparticles into colorectal cancer and normal cells. (**a**) Colorectal cancer HT29 and (**b**) Caco-2 cells and (**c**) normal CCD-112 cells were incubated with free doxorubicin (DOX), tHBcAg nanoparticles loaded with PAA-DOX (tHBcAg-PAA-DOX), folic acid (FA)-conjugated tHBcAg nanoparticles loaded with PAA-DOX (FA-tHBcAg-PAA-DOX), and FA-conjugated tHBcAg nanoparticles using the nanoglue and loaded with PAA-DOX (FA-N-tHBcAg-PAA-DOX) at equivalent DOX concentrations (5 μg/mL) at 37 °C and 5% CO_2_ for 1 h. The untreated cells were used as negative controls. The samples are labelled on the left. Nuclei were stained with Hoechst (blue), and DOX was excited at 480 nm. Scale bars indicate 20 μm.

Cellular uptake and localization of free DOX, tHBcAg-PAA-DOX, FA-tHBcAg-PAA-DOX and FA-N-tHBcAg-PAA-DOX in colorectal cancer and normal cells were visualized by live cell imaging microscopy, and the DOX uptake was quantified spectrophotometrically. Colorectal cancer HT29 and Caco-2 cells incubated with FA-tHBcAg-PAA-DOX exhibited a more intense red fluorescence in both the cytoplasm and nuclei, whereas cells incubated with free DOX showed a more intense red fluorescence in the nuclei (Fig. 7a and b). The fluorescence intensity in the cytoplasm increased considerably when the cells were incubated with FA-N-tHBcAg-PAA-DOX. This enhancement of DOX uptake could be attributed to a higher amount of FA conjugated to FA-N-tHBcAg-PAA-DOX using the nanoglue concept. Normal colorectal cells, CCD-112 cells, incubated with free DOX showed a stronger red fluorescence intensity compared with the same cells incubated with FA-tHBcAg-PAA-DOX and FA-N-tHBcAg-PAA-DOX (Fig. 7c). This demonstrates that the uptake of DOX by the normal cells was considerably reduced when the drug was packaged inside tHBcAg nanoparticles. Quantitative data also provided supporting evidence for higher uptake of DOX from FA-tHBcAg-PAA-DOX and FA-N-tHBcAg-PAA-DOX nanoparticles by HT29 and Caco-2 cells (Supplementary Figure S2a and b). HT29 and Caco-2 cells incubated with FA-tHBcAg-PAA-DOX and FA-N-tHBcAg-PAA-DOX showed a 3-fold and 4-fold higher DOX uptake, respectively, compared with the addition of DOX alone. However, the uptake of DOX by HT29 cells was higher than that of Caco-2 cells. In the normal cell line, CCD-112, the uptake of DOX was lower when it was packaged by tHBcAg (tHBcAg-PAA-DOX) and conjugated with FA (FA-tHBcAg-PAA-DOX and FA-N-tHBcAg-PAA-DOX) compared with the free DOX (Supplementary Figure S2c).

The cytotoxicity of various DOX formulations toward HT29, Caco-2 and CCD-112 cells was assessed using an MTT assay. The IC_50DOX_ values of free DOX, FA-tHBcAg-PAA-DOX and FA-N-tHBcAg-PAA-DOX for HT29 cells were 1.01 ± 0.10 μM, 0.22 ± 0.02 μM and 0.14 ± 0.01 μM, respectively (Supplementary Figure S3a). The IC_50DOX_ values of free DOX, FA-tHBcAg-PAA-DOX and FA-N-tHBcAg-PAA-DOX for Caco-2 cells were 59.00 ± 3.21 μM, 13.90 ± 1.12 μM and 8.10 ± 0.61 μM, respectively (Supplementary Figure S3b). This shows that FA-tHBcAg-PAA-DOX and FA-N-tHBcAg-PAA-DOX were more cytotoxic to HT29 and Caco-2 cells compared with free DOX. For normal colorectal cells, CCD-112 cells, the IC_50DOX_ values of free DOX, FA-tHBcAg-PAA-DOX and FA-N-tHBcAg-PAA-DOX were 1.10 ± 0.10 μM, 2.20 ± 0.20 μM and 2.02 ± 0.20 μM, respectively (Supplementary Figure S3c). This shows that the cytotoxicity of DOX against the normal cells was reduced when the drug was loaded inside tHBcAg nanoparticles. The tHBcAg nanoparticles, which served as a negative control, did not show any cytotoxic effects on the tested cancer cells or normal cells.

## Discussion

For effective delivery of an anticancer drug, pH-responsive nanoparticles are designed to store and protect the drug at physiological pH, and the drug is released when the pH trigger point is reached^29^,^30^. In this study, we developed pH-responsive VLNPs that can store DOX and possess a sustained-release property under tumour-associated conditions. A tumour-targeting ligand, FA, was therefore conjugated to the tHBcAg nanoparticles because it interacts strongly with the FR, which is highly expressed on the surface of many cancer cells, including colorectal cancer cells^16^,^25^,^31^.

In this study, a novel method to package DOX in tHBcAg nanoparticles was established. Like many viral capsids, tHBcAg nanoparticles are porous with a pore size of approximately 14 Å around the 3-fold axis and approximately 12–15 Å wide around the 2-fold axis^32^. The pores pose a challenge for packaging and retaining DOX inside the tHBcAg nanoparticles, as the drug can easily diffuse out from the particles. Therefore, DOX was mixed with PAA polymer, and they were packaged inside tHBcAg nanoparticles using a simple dissociation and association method.

Apart from functioning as a polymer for loading DOX into tHBcAg nanoparticles, PAA can also act as a pH-responsive nanovalve for controlled release of DOX from the nanoparticles. Here, we demonstrated that tHBcAg nanoparticles loaded with PAA and DOX retained DOX at physiological conditions (pH 7.4, 37 °C) and released the drug in acidic conditions (pH 5.4). These nanoparticles showed a sustained drug release profile *in vitro* at tumour tissue conditions (pH 5.4, 37 °C). The cumulative release of DOX in the tumour tissue conditions was significantly higher (P < 0.01) than that in physiological conditions (pH 7.4, 37 °C). The release of DOX in different pH environments can be explained by the hydrogen-bonding interaction between PAA and DOX. At pH 7.4, two types of hydrogen bonds form between the -COOH of PAA and –NH_2_ or –OH of DOX. The hydrogen-bonding interaction between DOX and PAA is the strongest at pH 7.4, resulting in an inefficient release^33^,^34^. Under mildly acidic conditions, the H^+^ competes with the hydrogen-bond-forming groups and weakens the hydrogen-bonding interactions^35^. Moreover, at low pH, DOX becomes more hydrophilic and water soluble; thus, more DOX is released from the tHBcAg-PAA-DOX nanoparticles into solution^28^,^34^,^36^. Because the microenvironment around tumour tissues, and in intracellular lysosomes and endosomes are slightly acidic^19^,^28^,^36^, DOX is released after being taken up by tumour tissues (pH 5–5.5).

A peptide that binds tightly with tHBcAg nanoparticles was used as a nanoglue to display CIPs for specific delivery of oligonucleotides and fluorescein into HeLa cells^37^. In this study, it is hypothesized that a tumour-targeting molecule can be displayed on the exterior of tHBcAg nanoparticles using this nanoglue for enhanced site-specific delivery of drugs into cancer cells. To confirm this concept, the pentadecapeptide (KKKGGGSLLGRMKGA), containing the nanoglue at the C-terminus and 3 Lys residues at the N-terminus, was synthesised and cross-linked to the spikes of tHBcAg nanoparticles loaded with DOX and PAA. FA molecules were then conjugated to the Lys residues to maximize their exposure and enhance site-specific targeting and drug delivery to colorectal cancer cells expressing the FR. TEM analysis revealed that packaging of DOX and PAA, cross-linking of the nanoglue and conjugation of FA had no adverse effects on the spherical structure of tHBcAg nanoparticles.

Accumulation of DOX in HT29 and Caco-2 cells increased considerably when they were treated with FA-tHBcAg-PAA-DOX and FA-N-tHBcAg-PAA-DOX nanoparticles compared with free DOX. This confirmed that the uptake of these nanoparticles by the colorectal cancer cells was *via* the FR. In addition, the uptake of FA-N-tHBcAg-PAA-DOX nanoparticles by these cells was higher than that of FA-tHBcAg-PAA-DOX nanoparticles. This finding can be explained by the fact that more FA molecules can be displayed on the surface of tHBcAg nanoparticles using the nanoglue, and therefore, this approach enhanced FR-mediated endocytosis by the cancer cells. However, the uptake of DOX by the normal colorectal cells, CCD-112 cells, was lower when the cells were treated with FA-tHBcAg-PAA-DOX and FA-N-tHBcAg-PAA-DOX nanoparticles.

The higher uptake of FA-tHBcAg-PAA-DOX and FA-N-tHBcAg-PAA-DOX by the cancer cells (HT29 and Caco-2) compared with the normal cells (CCD-112) can be explained by the fact that the FR is overexpressed in cancer cells^25^,^38^. The amount of FR on HT29 cells was demonstrated to be higher than that of Caco-2 cells^25^, which resulted in a higher DOX uptake by HT29 cells compared with Caco-2 cells. In addition, α-FR is highly expressed in cancer cells, while normal cells typically express β-FR^39^. The α-FR on cancer cells has a higher binding affinity for the free R-carboxylic acid exposed on the surface of FA-conjugated drug delivery systems^40^, whereas the β-FR on normal cells interacts tightly with the reduced form of FA, 5-methyltetrahydrofolate^41^,^42^. This is in line with our finding in which both the FA-tHBcAg-PAA-DOX and FA-N-tHBcAg-PAA-DOX nanoparticles were taken up at a much higher rate by HT29 and Caco-2 cells than CCD-112 cells.

The nanoglue is cross-linked at the tip of the spikes of tHBcAg nanoparticles, which is the outermost region of these particles. FA molecules are conjugated to the N-terminus of the nanoglue, and the flexibility of FA molecules may allow them to probe different regions of the cell surface and form multiple tethers between a FA-N-tHBcAg-PAA-DOX nanoparticle and a FR cluster. The affinity of such multivalent attachments can be several orders of magnitude higher than the interaction of a monovalent FA with a single receptor^43^; thus, a greater number of the FA-N-tHBcAg-PAA-DOX nanoparticles can internalize the cancer cells. At the same time, competition from endogenous folates for receptor occupancy *in vivo* will be reduced^43^. In addition, approximately 900 DOX molecules were loaded into each tHBcAg nanoparticle. Therefore, theoretically and practically, the amount of drugs delivered at each receptor will be amplified too. This considerably enhances the uptake of DOX by cancer cells.

A cytotoxicity test toward cancer and normal colorectal cells was performed to study the pharmacological activity of the packaged DOX. The results revealed that the FA-N-tHBcAg-PAA-DOX nanoparticles are more cytotoxic to HT29 and Caco-2 cells (IC_50DOX_ = 0.14 ± 0.01 μM and 8.10 ± 0.61 μM, respectively) compared with the other formulations. For both of the cancer cell lines (HT29 and Caco-2), the IC_50DOX_ of FA-tHBcAg-PAA-DOX and FA-N-tHBcAg-PAA-DOX formulations decreased by approximately 4-fold and 7-fold, respectively, compared with the IC_50DOX_ of free DOX. This shows that packaging of DOX in tHBcAg nanoparticles and conjugation of FA to the particles, particularly by using the nanoglue, increase the cytotoxicity of the packaged drug. Interestingly, packaging of DOX in either the FA-tHBcAg-PAA-DOX or FA-N-tHBcAg-PAA-DOX nanoparticles led to reduced cytotoxicity against the CCD-112 cells, indicating a conferred protection of the normal cells against the drug.

In summary, a novel method for packaging DOX inside tHBcAg nanoparticles and conjugation of FA to the particles using the nanoglue concept was established. The reactive amino groups on the Lys residues located at the N-terminus of the nanoglue cross-linked to the spikes of tHBcAg nanoparticles can be conjugated with tumour-targeting molecules. The conjugation of FA on tHBcAg nanoparticles using the nanoglue significantly enhanced (P < 0.01) the cellular uptake and accumulation of DOX in colorectal cancer HT29 and Caco-2 cells. Apart from FA, any tumour-targeting ligands can easily and rapidly be displayed on the exterior surface of VLNPs using the nanoglue, bypassing laborious, time-consuming, and tedious genetic engineering approaches. A sustained-release profile and a nearly complete release of all the packaged DOX in tumour tissue conditions render tHBcAg nanoparticles an attractive platform for pH-responsive drug delivery system.

## Methods

### Production and purification of tHBcAg nanoparticles

tHBcAg (residues 3–148) was produced in *E. coli* carrying the pR1-11E plasmid as described by Tan *et al*.^26^. The tHBcAg nanoparticles were purified by a fast protein liquid chromatography (FPLC) system (Akta Purifier; GE Healthcare) as described by Yoon *et al*.^44^. The purity of the tHBcAg was analysed with SDS-PAGE, and its concentration was determined using a Bradford assay^45^.

### Chemical cross-linking of peptide to tHBcAg nanoparticles

Chemical cross-linking of pentadecapeptide (KKKGGGSLLGRMKGA) to tHBcAg was performed by adding tHBcAg:peptide (1:4 ratio) to a mixture containing chemical modification reagents and phosphate buffer, as described by Lee *et al*.^37^ and Tang *et al*.^46^, with some changes. Briefly, tHBcAg (50 μg) and the peptide were incubated at 4 °C for 8 h in 100 μL of reaction buffer [25 mM NaH_2_PO_4_/Na_2_HPO_4_, 150 mM NaCl (pH 7.0), 1.8 mM Sulfo-NHS (N-hydroxysulfosuccinimide), and 1.8 mM EDC (1-ethyl-3-(3-dimethyl-aminopropyl)-carbodiimide hydrochloride)]. The cross linking of the pentadecapeptide to tHBcAg was analysed by SDS−PAGE, and the structure of tHBcAg nanoparticles was viewed under TEM.

### Conjugation of folic acid to tHBcAg nanoparticles using the nanoglue

FA (5 mg; Sigma-Aldrich), EDC (20 mg), and Sulfo-NHS (20 mg) were dissolved in sodium phosphate buffer (10 mM NaH_2_PO_4_/Na_2_HPO_4_, pH 6.0; 5 mL) containing DMSO [20% (v/v)] at room temperature (RT) for 4 h. The pH of the solution was then adjusted to 7.4 with NaOH, and the tHBcAg nanoparticles cross-linked with pentadecapeptide (3 mg) in sodium phosphate buffer (pH 7.4) were added. The mixture was agitated gently at 4 °C for 8–16 h and purified with sucrose density gradient ultracentrifugation (8–40%; 210,000 × *g*, for 5 h at 4 °C), as described by Tan *et al*.^26^. In a parallel experiment, activated FA was also added to tHBcAg nanoparticles, and the same conjugation procedure was followed. UV-visible measurement of tHBcAg nanoparticles and FA-conjugated tHBcAg nanoparticles at A_360_ was performed using a NanoDrop^TM^ 1000 spectrophotometer (Thermo Scientific), and the conjugated FA was quantified using an extinction coefficient of 5312 mol^−1^cm^−1^, as described by Ren *et al*.^22^. The conjugation efficiency (CE) of FA and the number of FA molecules conjugated to each nanoparticle (N_FA_) were calculated using equations 1 and 2, respectively.


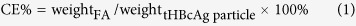



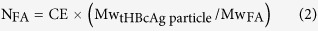


Mw represents the molecular weight.

### Transmission electron microscopy (TEM)

tHBcAg nanoparticles and derivatives (~0.25 mg/mL; 15 μL) were first adsorbed onto carbon coated grids (200 mesh) and then stained with freshly prepared uranyl acetate [2% (w/v)] for approximately 5 min. The samples were viewed under a TEM (100 kV; Hitachi H7700).

### Cell culture

The human colorectal cancer cell lines (HT29 and Caco-2) and normal cell line (CCD-112) were obtained from the American Type Culture Collection (ATCC). HT29 and Caco-2 cells were cultured in FA-deficient RPMI1640 medium (Gibco, Life Technologies), while the CCD-112 cells were grown in EMEM medium (ATCC) containing heat-inactivated foetal bovine serum (FBS, 10%). All cells were cultured at 37 °C in a humidified atmosphere of 5% CO_2_ and 95% air and were passaged twice weekly.

### Immuno-fluorescence microscopy

HT29 cells were seeded in a six-well plate (200,000 cells/well) containing sterile glass coverslips at 37 °C. After 24 h of incubation, different FA-conjugated tHBcAg preparations (25 μg) were added into the wells. The cells were incubated at 37 °C for 16 h, washed three times with the medium and fixed with paraformaldehyde (3.7%) in phosphate-buffered saline (PBS; 1.47 mM KH_2_PO_4_, 8.1 mM Na_2_HPO_4_, 137 mM NaCl, 2.7 mM KCl; pH 7.4) for 10 min. The cells were immediately permeabilized with ice-cold methanol at −20 °C for 6 min. Nonspecific binding was blocked by incubating the cells in blocking buffer (0.2 mg/mL BSA in PBS) for 1 h. The cells were incubated with rabbit anti-tHBcAg serum (1:200 dilutions in blocking buffer, 1 h at RT), followed by goat anti-rabbit IgG conjugated to Alexa Fluor^®^ 488 (1:1000 dilutions in blocking buffer; Life Technologies) in the dark for another 1 h. The cell nuclei were stained with Hoechst 33342 (Ex_360 nm_ and Em_460 nm_; 2 drops/mL PBS; Life Technologies) for 10 min. The cells were observed under a fluorescence microscope (Olympus America FSX100™).

### Packaging of DOX by tHBcAg nanoparticles

The method of dissociation and association, as described by Lee and Tan^13^, was used with some modifications, for packaging of DOX (Merck Millipore) by tHBcAg nanoparticles. Dissociation was performed by incubating the purified tHBcAg nanoparticles (1 mg/mL) in 2.5 M urea at 25 °C for 6–8 h. Polyacrylic acid (PAA, Mw = 450 KDa; 0.6 mg/mL; Sigma-Aldrich) was mixed with DOX (0.3 mg/mL) and incubated for 2 h at 25 °C. The PAA-DOX solution (1 vol) was then added to the dissociated tHBcAg nanoparticles (2 vol) and incubated for 1 h at 25 °C. The sample was dialysed against dialysis buffer (50 mM Tris–HCl, 100 mM NaCl, pH 8.0; 1 L, two times) at 4 °C to assemble the dissociated tHBcAg nanoparticles by removing the urea and excess DOX. The tHBcAg nanoparticles harbouring PAA-DOX were purified with sucrose density gradient ultracentrifugation (8–40%, w/v), as described by Tan *et al*.^26^, fractionated (400 μL per fraction), and analysed with a Bradford assay^45^, and the amount of DOX was measured at A_495_. tHBcAg nanoparticles loaded with DOX were then conjugated with FA using the nanoglue, as described earlier. The samples were observed with TEM.

### Loading efficiency of DOX and the number of DOX molecules packaged in tHBcAg nanoparticles

Sucrose fractions containing tHBcAg nanoparticles loaded with DOX were pooled and dialysed in dialysis buffer. After concentrating, loaded DOX was quantified at A_495_ (extinction coefficient, 8030 cm^−1^M^−1^). Loading efficiency (LE) of DOX and the number of DOX molecules packaged within each nanoparticle (N_DOX_) were calculated using equations 3 and 4, respectively^22^,^47^.


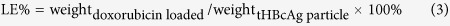



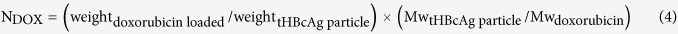


Mw represents the molecular weight.

### Native agarose gel electrophoresis (NAGE)

The migration profiles of the tHBcAg nanoparticles loaded with DOX were studied by NAGE. The samples were electrophoresed on an agarose gel [1.5% (w/v)] in TBE buffer (90 mM Tris–borate, 2 mM EDTA; pH 8.3) for 3 h at 80 V at RT. DOX bands were visualized by ultraviolet (UV) illumination using a GelDoc 2000 Imaging System (Bio-Rad), while protein bands were stained with Coomassie brilliant blue (CBB).

### *In vitro* release of DOX from tHBcAg nanoparticles

A drug release experiment was conducted using the dialysis method, as described by Ren *et al*.^22^, with some modifications. tHBcAg-PAA-DOX, FA-tHBcAg-PAA-DOX and FA-N-tHBcAg-PAA-DOX samples (2 mL, equivalent to 250 μg/mL DOX) were placed in individual dialysis tubes (MWCO 12 KDa, Sigma-Aldrich). The samples were dialysed against PBS buffer (50 mL) at pH 7.4 and pH 5.4, separately, with gentle and constant stirring at 37 °C. At specific time points, the release buffer (1 mL) was collected and A_495_ was measured to quantify the released DOX. Each time, the collected buffer was replaced with fresh medium (1 mL). Dialysis of free DOX (250 μg/mL) in the same conditions was included as a control.

### Live-cell imaging

HT29 and Caco-2 cells (2.0 × 10^5^ cell/mL) and CCD-112 cells (1.0 × 10^5^ cell/mL) were sub-cultured in 6-well plates. After 24 h, each medium was replaced with fresh medium (1 mL) containing free DOX, tHBcAg-PAA-DOX, FA-tHBcAg-PAA-DOX and FA-N-tHBcAg-PAA-DOX with a constant concentration of DOX (5 μg/mL), and the cells were incubated for 1 h. The cells were washed six times with PBS (pH 7.4) and fixed with paraformaldehyde [3.7% (w/v), in PBS (pH 7.4)] for 10 min at 25 °C. Cell nuclei were stained with Hoechst 33342. Images of the stained substrates were taken using an Olympus Live Cell Imaging (EX_480 nm_ and Em_535 nm_) microscope.

### Cytotoxicity of DOX packaged in tHBcAg nanoparticles

The cytotoxicity of DOX, tHBcAg-PAA-DOX, FA-tHBcAg-PAA-DOX, and FA-N-tHBcAg-PAA-DOX was determined using an MTT cell viability assay on colorectal cancer (HT29 and Caco-2) and normal cell (CCD-112) lines. The HT29, Caco-2 and CCD-112 cells were seeded in 96-well plates at a density of 10000, 20000 and 7000 cells/well, respectively. After 24 h, the culture media were aspirated, and the cells were treated with different concentrations of free DOX or DOX formulations in fresh media (0.01–10.00 μM for HT29 and CCD-112 cells; 0.01–100.00 μM for Caco-2 cells) for 3 h. The media containing DOX or different formulations were replaced with fresh media without DOX, and the cells were incubated for 96 h. Then, the MTT was added and, after 4 h of incubation, A_570_ was measured using a Uquant ELISA plate reader (BioTeck Instruments). The cytotoxicity of tHBcAg was studied as a negative control.

### Statistical analysis

Statistical analysis was performed using the SPSS programme. Values of p < 0.01 were considered significant.

## Additional Information

**How to cite this article**: Biabanikhankahdani, R. *et al*. pH-responsive Virus-like Nanoparticles with Enhanced Tumour-targeting Ligands for Cancer Drug Delivery. *Sci. Rep.* **6**, 37891; doi: 10.1038/srep37891 (2016).

**Publisher's note:** Springer Nature remains neutral with regard to jurisdictional claims in published maps and institutional affiliations.

## Supplementary Material

Supplementary Information
