# Supplementary material for: pH-responsive Virus-like Nanoparticles with Enhanced Tumour-targeting Ligands for Cancer Drug Delivery
Source: Sci Rep. 2016 Nov 24;6:37891. doi: 10.1038/srep37891 (PMC5121657; doi:10.1038/srep37891)
Supplement: Supplementary Information [file srep37891-s1.pdf]

Supplementary Information

pH-responsive Virus-like Nanoparticles with Enhanced Tumour-targeting Ligands for Cancer Drug Delivery

Roya Biabanikhankahdani[1], Noorjahan Banu Mohamed Alitheen[2,4], Kok Lian Ho[3], Wen Siang Tan[*1,4]

[1]Department of Microbiology and [2]Department of Cell and Molecular Biology, Faculty of Biotechnology and Biomolecular Sciences, Universiti Putra Malaysia, 43400 UPM Serdang, Selangor, Malaysia. [3]Department of Pathology, Faculty of Medicine and Health Sciences, Universiti Putra Malaysia, 43400 UPM Serdang, Selangor, Malaysia. [4]Institute of Bioscience, Universiti Putra Malaysia, 43400 UPM Serdang, Selangor, Malaysia

Methods

Fluorescein labeling of the tHBcAg nanoparticles

The primary amine groups of Lys residues of tHBcAg at positions 7 and 96 were labelled using the amine-selective NHS-fluorescein reagent (Thermo Scientific, Rockford, IL, USA). The labeling procedure was performed as recommended by the manufacturer. The non-reacted NHS-fluorescein molecules were removed by dialysis against sodium phosphate buffer (25 mM NaH_2PO_4/Na_2HPO_4, pH 7.4) containing NaCl (150 mM) at 4 °C. In this experiment, the labeling reaction was performed at pH 7.4 to avoid the protonation of the α-amine at the N-terminal end of tHBcAg which limits the labeling of fluorescein molecules at the ε-amine of the Lys side chains. Fluorescein-labelled tHBcAg nanoparticles were cross-linked with the pentadecapeptide and then conjugated with folic acid (FA) as described earlier.

Cellular uptake of doxorubicin

HT29, Caco-2 and CCD-112 cells were seeded in six-well plates (10^5 cells per well) and incubated for 24 h. Then, free doxorubicin (DOX), tHBcAg nanoparticles loaded with PAA-DOX (tHBcAg-PAA-DOX), folic acid (FA)-conjugated tHBcAg nanoparticles and loaded with PAA-DOX (FA-tHBcAg-PAA-DOX), and FA-

conjugated tHBcAg nanoparticles using the nanoglue and loaded with PAA-DOX (FA-N-tHBcAg-PAA-DOX) were added at equivalent DOX concentration (5 μg/mL). The excess sample was removed by washing three times with ice-cold PBS (pH 7.4) and the cells were then lysed with lysis buffer [50 mM Tris (pH 7.4), 0.8% Triton, 0.2% SDS] in a total volume of 0.5 mL per well. Internalized DOX was measured at A_{490} by using a microplate reader (ELX 800; BioTeck Instruments).

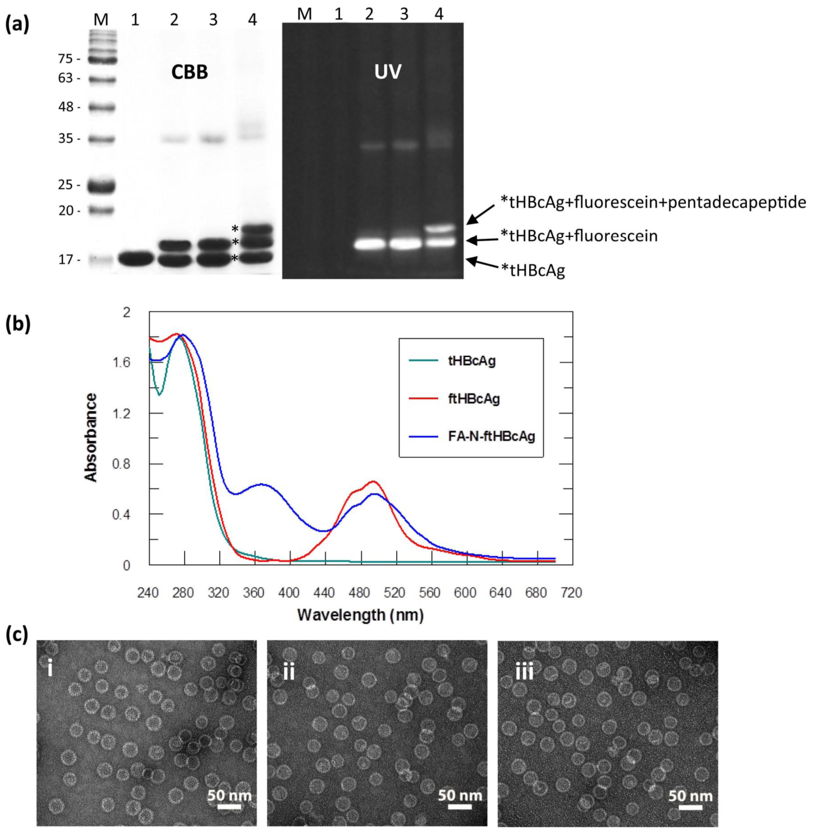

Supplementary Figure S1. Conjugation of folic acid to fluorescein-labelled tHBcAg nanoparticles using the nanoglue concept. (a) Chemical cross-linking of the pentadecapeptide to fluorescein-labelled tHBcAg (ftHBcAg) nanoparticles. The samples were separated on an SDS-polyacrylamide gel and stained with Coomassie brilliant blue (CBB) and observed under ultraviolet (UV) illumination. Lanes M, molecular mass markers (kDa); 1, tHBcAg control; 2, ftHBcAg; 3, ftHBcAg plus cross-linkers; and 4, ftHBcAg plus pentadecapeptide and cross-linkers. Asterisks and

arrows indicate the protein bands of tHBcAg, tHBcAg+fluorescein (ftHBcAg), and tHBcAg+fluorescein+pentadecapeptide (N-ftHBcAg). SDS-PAGE and UV visualization confirmed that the tHBcAg was labelled with fluorescein and cross-linked with the pentadecapeptide (b) Conjugation of folic acid (FA) to tHBcAg using the nanoglue. Spectra of tHBcAg nanoparticles (tHBcAg), fluorescein-labelled tHBcAg nanoparticles (ftHBcAg) and fluorescein-labelled tHBcAg nanoparticles conjugated with FA using the nanoglue (FA-N-ftHBcAg). (c) Electron micrographs of tHBcAg nanoparticles. Nanoparticles formed by tHBcAg (i), ftHBcAg (ii) and FA-N-ftHBcAg (iii). White bars indicate 50 nm.

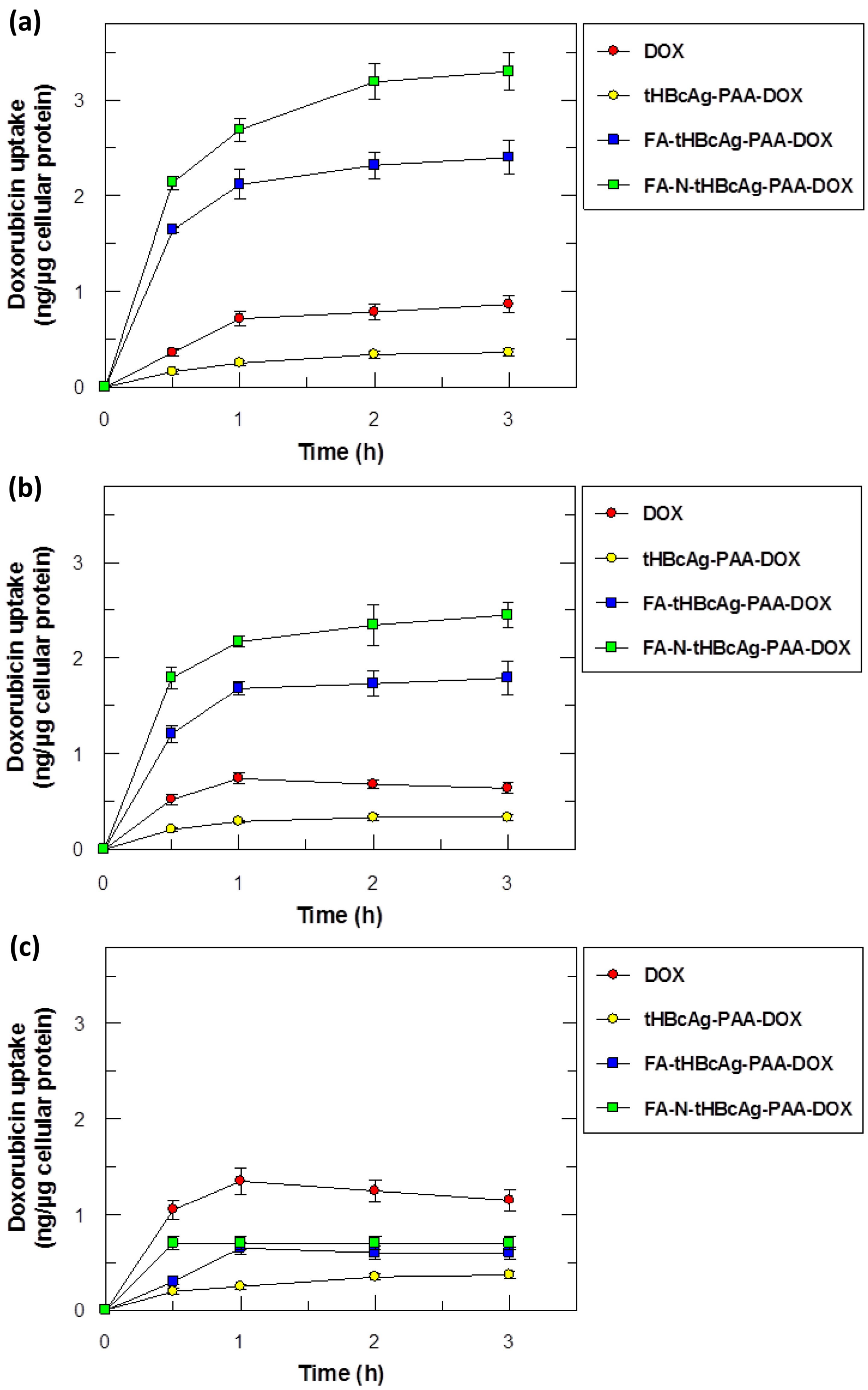

Supplementary Figure S2. Doxorubicin uptake by colorectal cancer and normal cells. Cellular uptake of doxorubicin by (a) cancer HT29, (b) Caco-2 and (c) normal CCD-112 cells incubated with free doxorubicin (DOX), tHBcAg nanoparticles loaded with PAA-DOX (tHBcAg-PAA-DOX), folic acid (FA)-conjugated tHBcAg

nanoparticles and loaded with PAA-DOX (FA-tHBcAg-PAA-DOX) and FA-conjugated tHBcAg nanoparticles using the nanoglue and loaded with PAA-DOX (FA-N-tHBcAg-PAA-DOX). Data represent mean ± SD of triplicate determinations.

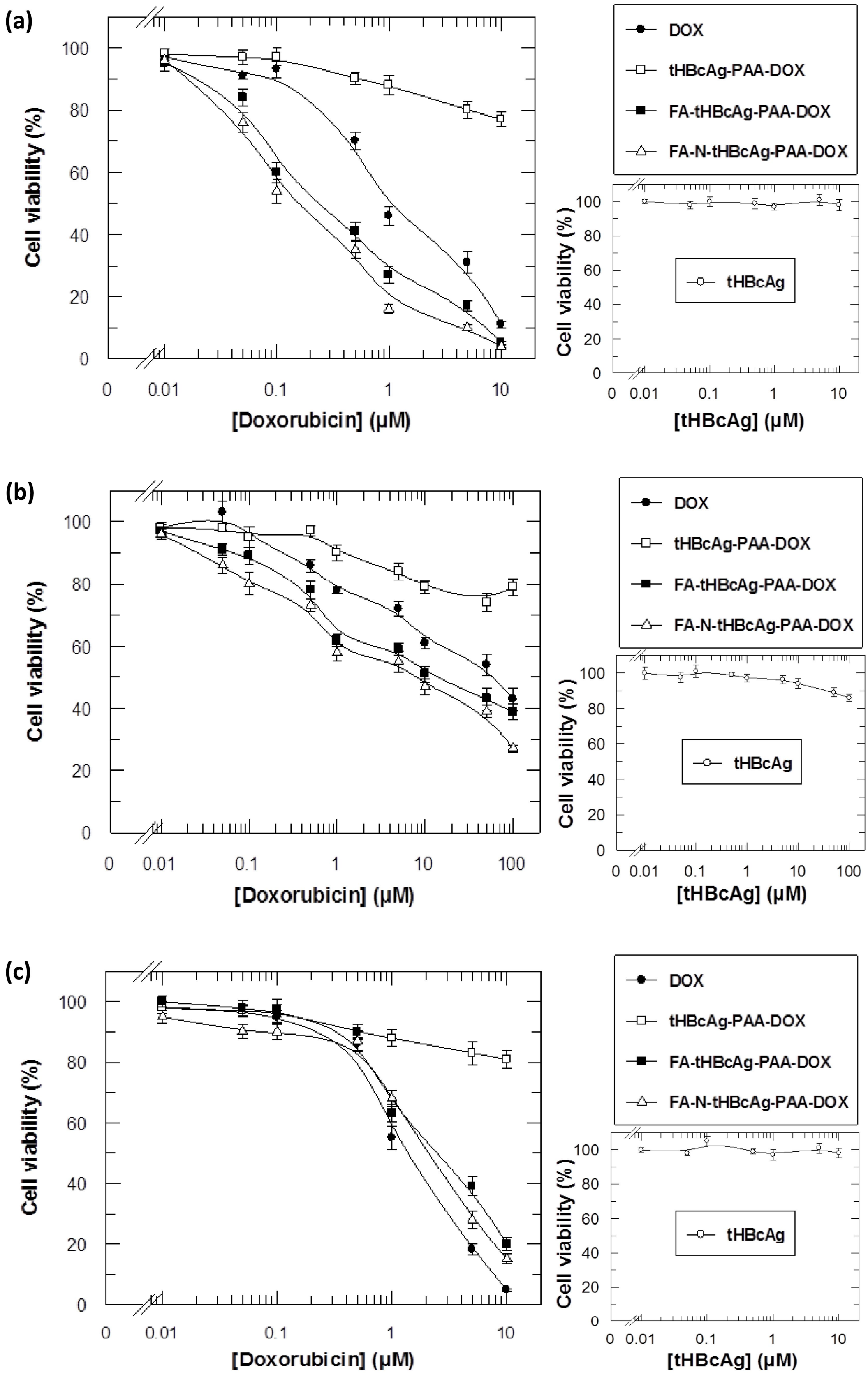
(a)
Cell viability (%)
[Doxorubicin] (μM)
DOX
tHBcAg-PAA-DOX
FA-tHBcAg-PAA-DOX
FA-N-tHBcAg-PAA-DOX
[tHBcAg] (μM)
tHBcAg
(b)
Cell viability (%)
[Doxorubicin] (μM)
DOX
tHBcAg-PAA-DOX
FA-tHBcAg-PAA-DOX
FA-N-tHBcAg-PAA-DOX
[tHBcAg] (μM)
tHBcAg
(c)
Cell viability (%)
[Doxorubicin] (μM)
DOX
tHBcAg-PAA-DOX
FA-tHBcAg-PAA-DOX
FA-N-tHBcAg-PAA-DOX
[tHBcAg] (μM)
tHBcAg

Supplementary Figure S3. Viability of colorectal cancer and normal cells after being treated with various doxorubicin formulations at equal doxorubicin concentration. Cell viability of colorectal cancer HT29 (**a**), Caco-2 (**b**) and normal CCD-112 (**c**) cells. Folic acid (FA)-conjugated tHBcAg nanoparticles using the nanoglue and loaded with PAA-DOX (FA-N-tHBcAg-PAA-DOX) was more efficient in inhibiting the growth of HT29 and Caco-2 cells than other formulations. In contrast, packaging of doxorubicin (DOX) in the FA-conjugated tHBcAg nanoparticles led to decrease cytotoxicity on normal CCD-112 cells, resulted in protection of the normal cells against DOX. Data represent mean ± SD of triplicate determinations. Small graphs on the right show that tHBcAg nanoparticles (tHBcAg) are not toxic to the tested cancer and normal cells.
